# Supplementary material for: Identification of Freezing-Responsive microRNAs and Their Targets in Chinese Jujube by Small RNA and Degradome Sequencing
Source: Int J Mol Sci. 2026 Sep 4;27(17):7912. doi: 10.3390/ijms27177912 (PMC13566890; doi:10.3390/ijms27177912)
Supplement: Supplementary file 1 [file ijms-27-07912-s001.zip › Supplementary Material S2/novel_miR_136.pdf]

Provisional ID	:	novel_miR_136
Score total	:	980.9
Score for star read(s)	:	3.9
Score for read counts	:	974.4
Score for mfe	:	1
Score for randfold	:	1.6
Score for cons. seed	:	
Total read count	:	1923
Mature read count	:	1920
Loop read count	:	0
Star read count	:	3

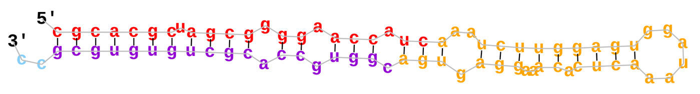

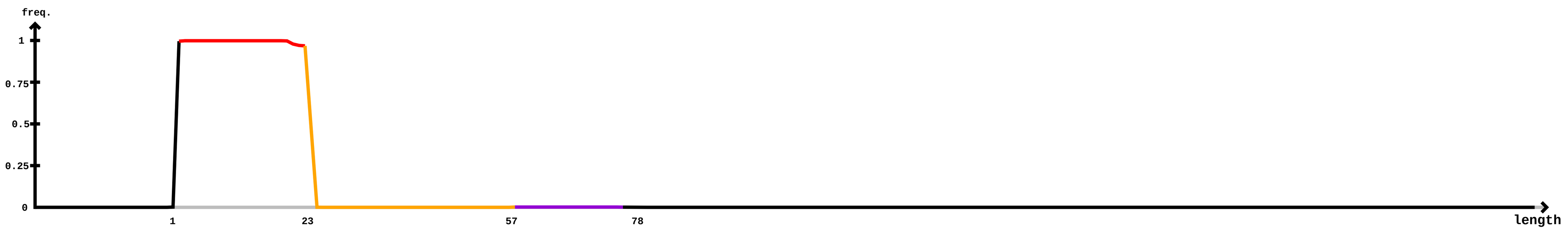

Mature **Star**

```
5'- gaaguagaggaggggguuuugggcgcacgcuagcggggaaccaucaaaucuuggaguggauaaacucacaaaggagugacggugccacgcugugugcgccucaaaugccuuucucucgcugauuucuauuccauuuuaugccgcucuuuauauuauuguuggaagauaaagaaaaaagguaaucaacuacuuuccuuucaauuuuuauuuuuaaaaaaaauaaaauaaaaaugaacccaucacuccuuugc   -3'   obs
    gaaguagaggaggggguuuugggcgcacgcuagcggggaaccaucaaaucuuggaguggauaaacucacaaaggagugacggugccacgcugugugcgccucaaaugccuuucucucgcugauuucuauuccauuuuaugccgcucuuuauauuauuguuggaagauaaagaaaaaagguaaucaacuacuuuccuuucaauuuuuauuuuuaaaaaaaauaaaauaaaaaugaacccaucacuccuuugc         exp
    ...((((((((((((((.((((((((((.((((.((.(((.(((..((((((((.....)))).))..))).))).))).)).))))))))))))))))....(((((((...((..((.((((((..(.((..((((.......))))..)).).)))))).))..))..))))))).................((((((((((.........))))))))))))))))....)))))))))   reads   mm   sample
    ....................Ccgcacgcuagcggggaaccauc.......................   1     1   JCK-1
    ....................Ccgcacgcuagcggggaaccauc.......................   2     1   JCK-2
    .....................cgcacgcuagcggggaac...........................   2     0   LT-3
    .....................cgcacgcuagcggggaacc..........................   10    0   JCK-2
    .....................cgcacgcuagcggggaacc..........................   2     0   LT-2
    .....................Ugcacgcuagcggggaacc..........................   1     1   JCK-2
    .....................cgcacgcuagcggggaacc..........................   9     0   JCK-3
    .....................cgcacgcuagcggggaacc..........................   3     0   LT-3
    .....................cgcacgcuagcggggaacc..........................   3     0   LT-1
    .....................cgcacgcuagcggggaacc..........................   8     0   JCK-1
    .....................cgcacgcuagcggggaacca.........................   2     0   LT-2
    .....................cgcacgcuagcggggaacca.........................   12    0   JT-1
    .....................cgcacgcuagcggggaacca.........................   2     0   LT-3
    .....................cgcacgcuagcggggaaccau........................   4     0   LT-1
    .....................cgcacgcuagcggggaaccau........................   1     0   JCK-1
    .....................cgcacgcuagcggggaaccauc.......................   12    0   JT-3
    .....................cgcGcgcuagcggggaaccauc.......................   1     1   JCK-1
    .....................cgUacgcuagcggggaaccauc.......................   2     1   JCK-3
    .....................cgcacgcuagcggggaaccauc.......................   15    0   LT-2
    .....................cgcacgcuagcNgggaaccauc.......................   1     1   JCK-1
    .....................cgcacAcuagcggggaaccauc.......................   1     1   LT-2
    .....................cgcacgcuCgcggggaaccauc.......................   3     1   JCK-2
    .....................cgcacgcuagcggggaaccauA.......................   1     1   LT-1
    .....................cgcaAgcuagcggggaaccauc.......................   1     1   JCK-3
    .....................cgcacgcuagcggggaaccauA.......................   1     1   LT-2
    .....................cgcacgcuagcggggaaccUuc.......................   1     1   LT-3
    .....................cgcacgcuagcggggaaccauc.......................   637   0   JCK-2
    .....................cgcacgcuagcggggaaccauU.......................   10    1   LT-2
    .....................cgcacgcuagcggggaaccauc.......................   529   0   JCK-3
    .....................cgcacgcuagcggggaaccauc.......................   24    0   LCK-3
    .....................cgcacgcuagcggggaaccauc.......................   3     0   JT-2
    .....................cgcaUgcuagcggggaaccauc.......................   1     1   JCK-3
    .....................cgcacgcuagcggggaaccauU.......................   2     1   JCK-1
```

Mature **Star**

```
gaaguagaggaggguuuugggcgcacgcuagcggggaaccaucaaaucuuggaguggauaaacucacaaaggagugacggugccacgcugugugcgccucaaaugccuuucucucgcugauuucuauuccauuuuaugccgcucuuuauauuauuguuggaagauaaagaaaaaagguaaucaacuacuuuccuuucaauuuuuauuuuuaaaaaaaauaaaauaaaaaugaacccaucacuccuuugc
.....................cgcacgcuagcCgggaaccauc..............................................................................................................................................................................................................   1     1    JCK-1
.....................cgcacgcuagcAgggaaccauc..............................................................................................................................................................................................................   1     1    JCK-1
.....................cNcacgcuagcggggaaccauc..............................................................................................................................................................................................................   1     1    JCK-2
.....................cgcacgcuagcggggaaccauU..............................................................................................................................................................................................................   1     1    JCK-3
.....................cgcacgcuagcggggaaccauc..............................................................................................................................................................................................................   35    0    LT-1
.....................cgcacgcuCgcggggaaccauc..............................................................................................................................................................................................................   1     1    JCK-3
.....................cgcacgcuagcggggaaccauU..............................................................................................................................................................................................................   1     1    LT-3
.....................cgcacgcuaAcggggaaccauc..............................................................................................................................................................................................................   1     1    JCK-1
.....................cgcacgcuagcggggaaccauU..............................................................................................................................................................................................................   2     1    LT-1
.....................cgcacgcuagcggggaaccauc..............................................................................................................................................................................................................   420   0    JCK-1
.....................cgcacgcuagcggggaaccCuc..............................................................................................................................................................................................................   1     1    JCK-1
.....................cgcacgcuagcggggaaccauU..............................................................................................................................................................................................................   5     1    JCK-2
.....................cgcacgcuagcggggaaccauc..............................................................................................................................................................................................................   48    0    LT-3
.....................cgcacgcuCgcggggaaccauc..............................................................................................................................................................................................................   2     1    JCK-1
.....................cNcacgcuagcggggaaccauc..............................................................................................................................................................................................................   2     1    JCK-1
.....................cgcCcgcuagcggggaaccauc..............................................................................................................................................................................................................   1     1    JCK-1
.....................cgcacgcuagcggggaaccaucU.............................................................................................................................................................................................................   3     1    LT-1
.....................cgcacgcuagcggggaaccaucU.............................................................................................................................................................................................................   11    1    JCK-1
.....................cgcacgcuagcggggaaccauca.............................................................................................................................................................................................................   9     0    JCK-3
.....................cgcacgcuagcggggaaccaucU.............................................................................................................................................................................................................   6     1    LT-3
.....................cgcacgcuagcggggaaccaucU.............................................................................................................................................................................................................   31    1    JCK-2
.....................cgcacgcuagcggggaaccaucU.............................................................................................................................................................................................................   15    1    JCK-3
.....................cgcacgcuagcggggaaccauca.............................................................................................................................................................................................................   7     0    JCK-2
.....................cgcacgcuagcggggaaccauca.............................................................................................................................................................................................................   2     0    JCK-1
.....................cgcacgcuagcggggaaccauUaa............................................................................................................................................................................................................   1     1    JCK-3
......................gcacgcuagcggggaaccauc..............................................................................................................................................................................................................   3     0    LT-3
......................gcacgcuagcggggaaccauc..............................................................................................................................................................................................................   2     0    JCK-2
.............................................................................cggugccacgcugugugc..........................................................................................................................................................   1     0    LT-1
.............................................................................cggugccacgcugugugcg.........................................................................................................................................................   1     0    JCK-2
.............................................................................cggugccacgcugugugcg.........................................................................................................................................................   1     0    JCK-1
```
